# The Use of Antidepressant Drugs in Climacteric Syndrome

**DOI:** 10.1055/s-0040-1701457

**Published:** 2020-01

**Authors:** Maria Célia Mendes, Marcos Felipe Silva de Sá

**Affiliations:** 1 Department of Gynecology and Obstetrics, Faculdade de Medicina, Universidade de São Paulo, Ribeirão Preto, SP, Brazil

Vasomotor symptoms (VMS) or hot flashes interfere with women's quality of life and are the probable cause of sleep disorders, lack of energy, depression and tiredness in the peri- and postmenopausal period. These symptoms normally last between 2 and 10 years, with an average of 7.4 years or more.^1^ ^2^ Estrogen therapy is the treatment of choice for VMS and reduces both the weekly frequency and the severity of these symptoms.^3^ ^4^ For hot flashes relief, hormone treatment lasts 3 to 5 years and discontinuity may lead to recurrence in up to 50% of symptoms. On the other hand, by considering the benefits of hormone therapy for osteoporosis prevention, quality of life improvement and treatment of persistent VMS,^1^ there is a current trend to extend treatment until the age of 60 or 65 years old.

Other drug therapies are suggested for women who do not wish to undergo estrogen therapy, usually for fear of cancer, and those with contraindications to hormone treatment, although the results of these therapies are far lower than conventional estrogen therapy. These include selective serotonin reuptake inhibitors (SSRIs) and selective serotonin and norepinephrine reuptake inhibitors (SNRIs). Despite the inferior therapeutic results, after estrogen, these are the most used drugs for the treatment of VMS,^5^ and they have a very fast action (in days) in reducing hot flashes, while their antidepressant action will occur later (in weeks).^6^

The efficacy of this treatment is hard to evaluate, because the symptom reduction may be caused by the placebo effect of these drugs.^5^ Furthermore, clinical trials have no long-term follow up of patients, and most studies evaluate treatment efficacy by comparing with placebo at 4 to 12 weeks and the effect at 12 to 24 weeks after drug discontinuation.^7^ Both SSRIs and SNRIs bring mild to moderate improvement in symptoms and 25% to 69% reduction in hot flashes.^7^ ^8^ ^9^ For the treatment of VMS, the North America Menopause Society (NAMS) recommends paroxetine (recommendation level I), citalopram, escitalopram, venlafaxine and desvenlafaxine (level II),^7^ although only paroxetine has been approved by the FDA and is recommended by the American College of Obstetrics and Gynecology (ACOG).^10^

Regarding fluoxetine and sertraline, publications present conflicting results. Some authors argue that these medications are less effective and should be considered as a second line treatment.^11^ In some studies, less consistent results were observed with no statistically significant improvement in hot flashes.^7^ In contrast, other studies have shown a reduction in VMS,^12^ ^13^ including in women with breast cancer.^14^ ^15^ For these reasons, prescriptions are recommended in various services,^16^ and in Brazil these medications are provided for free by the Ministry of Health; hence, they are more accessible to the entire population, especially those with low purchasing power.

Regarding adverse events, in a systematic review and meta-analysis published in 2014, no difference was found between the most cited side effects when comparing SSRIs with the placebo group.^17^ However, in several other studies, nausea, dry mouth, constipation, headache, and loss of appetite were the most frequently reported side effects with the use of SSRI/SNRI.^9^ ^18^ ^19^ Anorexia, vomiting, sexual dysfunction and insomnia^19^ or improved sleep were also reported with use of with paroxetine.^20^ ^21^ ^22^

Sexual dysfunction caused by SSRIs/SNRIs occurs in 32.5% to 73% of patients.^23^ ^24^ According to some authors, sexual dysfunction appears to be more related to medication dose or prior depression.^25^ Since increased blood pressure is a side effect that may arise with the use of SNRIs, there should be caution in the use by hypertensive patients,^26^ and these drugs are not recommended as a first line treatment in hypertensive women.^27^ Nowadays, the rise in antidepressant prescriptions has been a cause for much concern worldwide. In France, the overall prevalence of prescriptions increased from 6.5% in 1999–2000 to 10.4% in 2009–2010^28^ and in the US, from 5.84% in 1996 to 10.52% in 2005.^29^ In the Netherlands, the use of these drugs almost doubled between 1996 and 2012^30^ and in the United Kingdom, from 1995 to 2011, prescriptions increased from 61.9% to 129.9% per 1,000 people-year.^31^

In the Netherlands, between 1996 and 2012, long-term therapy was higher among women than men (two thirds of patients) with predominance in the age group of 45 to 64 years old (45% of them).^30^ Selective serotonin reuptake inhibitors accounted for 52% of prescribed antidepressants, and among prescriptions in general, 47% were for depression, 23% for anxiety and ~ 25% for somatic reasons (ill defined).^31^ This age group covers both peri- and postmenopausal patients and it is very likely that women with vasomotor and neurovegetative symptoms characteristic of this climacteric phase were included, as anxiety and depression are often associated with hot flashes.

However, the prolonged and justified use for estrogen therapy does not apply to alternative SSRI or SNRI therapy for the treatment of postmenopausal women. According to some authors, there is no conclusive evidence on the safety of antidepressants over time and their use could be more dangerous than beneficial, because it could interfere with the adaptive processes regulated by serotonin.^32^ The menopausal transition is an adaptive process of physiological mechanisms exerted by serotonergic neurons that are “poorly regulated” in this period, as a result of the estrogen level drop.^33^ After some period of hormonal instability, there is a re-adaptation of the organism to the new hypoestrogenic level, and hot flash symptoms and its repercussions on the female organism disappear. As SSRIs would be indicated to restore that balance, they should be prescribed for the shortest possible time.

Therefore, some questions arise: how often do doctors offer the discontinuation of SSRI or SNRI therapy when the patient reports being well after the start of medication? Is there any control over the duration of the use of these drugs? The literature on the use of SSRIs/SNRIs in climacteric women addressing this aspect of therapy is scarce. Prolonged use of these drugs may result in ineffectiveness and possible risks. In women, especially older women, are reported higher risks for falls and fractures, stroke, suicide attempts, epileptic seizures and digestive bleeding.^34^ According to the literature, two thirds of outpatients with anxiety and/or depression receive treatment with psychotherapy, notably antidepressants, and these are generally used for long periods.^35^ In the Netherlands, 30% of patients taking antidepressants do so for at least one year; in England, half of patients and in the USA, two thirds use the medication for at least two years. Only 10% of the patients discontinue the use of these drugs each year.^36^ ^37^ ^38^ ^39^

With regard to climacteric symptoms, information on overprescription of these drugs is not conclusive. Literature data specifically focused on the time of use and monitoring of patients receiving this treatment for climacteric VMS are frustrating. Side effects of antidepressants are underreported in the literature because they result from short-term studies. Thus, gynecologists who treat women in the climacteric period should be alert to common and persistent side effects with long-term use.^40^ When treating climacteric VMS, the most rational should be the use for short periods of time. When SSRIs or SNRIs are prescribed, patients should return in short time intervals for an initial assessment of therapeutic outcomes and side effects.

According to international consensus, the discontinuity of antidepressants should be addressed at six to 18 months after symptom remission in case of anxiety and four to 12 months in case of depressive disorders. Unnecessary continuation of antidepressant use may result in severe side effects and harm the health of patients.^34^ Therefore, the recommendation is an individualized treatment based on international guidelines.^41^ ^42^

For the treatment of hot flashes, unfortunately, there are no protocols that clearly determine how long SSRIs/SNRIs can or should be used in climacteric women. In the absence of evidence, patients who would eventually benefit from relief of depressive symptoms in the perimenopause may be reluctant to discontinue therapy for fear of symptom recurrence. Thus, many patients with transient episodes of depression or anxiety resulting from vasomotor phenomena receive antidepressant therapy at the beginning of treatment and prolong it beyond the necessary time, thereby becoming dependent on this therapy, which is often unnecessary and dispensable.^43^ ^44^

This question is not intended to restrict the prescription of such drugs, as they are relatively safe products. In Brazil, they are not even included in the group of controlled drugs; hence, far from controlled, addictive drugs, which facilitates the use and prescription. However, the increasing use of antidepressants is worrisome, not because of the increase in indications and prescriptions for new patients, but mainly due to the prolonged use by those already taking the drug. Long-term use is advisable only in cases of chronicity or in patients who experience recurrence of symptoms after withdrawal. In such situations, and if associated with complaints of depression and anxiety, support from psychiatric specialists is advised for the benefit of the patient.

Despite much controversy, SSRIs/SNRIs are yet another therapeutic option for treating hot flashes, although the results are not exciting in most patients. For women who cannot or do not wish to take estrogens, non-hormonal management, such as SSRI or SNRI is a realistic and safe therapeutic option^45^ as long as proper precautions are taken to avoid unnecessary prolonged use.
